# Exercise Addiction and Muscle Dysmorphia: The Role of Emotional Dependence and Attachment

**DOI:** 10.3389/fpsyg.2021.681808

**Published:** 2021-06-18

**Authors:** Leticia Olave, Ana Estévez, Janire Momeñe, Roger Muñoz-Navarro, María José Gómez-Romero, María Jesús Boticario, Itziar Iruarrizaga

**Affiliations:** ^1^Department of Experimental Psychology, Cognitive Processes and Speech Therapy, Complutense University of Madrid, Madrid, Spain; ^2^Department of Personality, Evaluation and Psychological Treatment. University of Deusto, Bilbao, Spain; ^3^Department of Psychology and Sociology, University of Zaragoza, Zaragoza, Spain; ^4^Stress and Health Research Group, School of Psychology, Autonomous University of Barcelona, Barcelona, Spain; ^5^Egarsat, Terrassa, Spain; ^6^Cognition, Emotion and Health, Research Group, Madrid, Spain

**Keywords:** emotional dependence, behavioral addictions, excessive exercising, attachment dimensions, muscle dysmorphia

## Abstract

**Background:** Both exercise addiction (EA) and muscle dysmorphia (MD) primarily involve the compulsive practice of physical exercise and are classified as behavioral addictions in different lines of research. These types of addictions are frequently comorbid with other addictive pathologies, such as emotional dependence (ED), which is closely related to childhood attachment. This study is presented to address the scarcity of research relating EA and MD with other behavioral addictions. The aims are to analyze the sex differences found in emotional dependence, attachment dimensions, EA and MD; to analyze the association between EA and MD and other behavioral addictions, such as emotional dependence and attachment dimensions; and to analyze the possible role that childhood attachment plays in mediating the interaction between emotional dependence and EA and MD.

**Method:** The sample comprised 366 participants (54.6% women) aged 17–31 (M = 23.53; SD = 6.48).

**Results:** There are sex differences, with men scoring higher in EA, MD, and ED. The positive relationship between EA and ED (values between 0.16 and 0.28), MD (presenting values between 0.42 and 0.70), and attachment styles based on preoccupation, interference, permissiveness, and value of parental authority (values between 0.11 and 0.14) is highlighted. On the other hand, MD was positively related to ED (values ranging from 0.24 to 0.36) and attachment styles based on the value of parental authority, self-sufficiency, and resentment toward parents (between 0.17 and 0.18), and negatively related to secure attachment (values between −0.13 and −0.18). Likewise, the predictive role of ED and attachment styles in EA and MD was tested. And it was observed how attachment styles mediated the relationship between EA and ED, as well as the relationship between MD and ED. Thus, it has been shown that people with EA and MD present attachment styles that may influence negatively the decision-making process when choosing inappropriate strategies to achieve adequate emotional regulation, even selecting inappropriate goals for physical exercise with negative consequences.

**Conclusions:** The dissemination of the findings among mental health and sports science professionals is necessary to develop prevention and intervention strategies for people affected by EA and MD.

## Introduction

Exercise addiction (EA) is a type of behavioral addiction and is characterized by a loss of behavioral control resulting in compulsive exercising (Hausenblas and Downs, 2002a; Scharmer et al., 2020). Initially, this conduct is driven by positive reinforcement, and, later, it persists due to negative reinforcement (Echeburúa et al., 2009). According to Griffiths (1997) and Terry et al. (2004), EA is related to the concepts of abstinence, tolerance, relevance (importance that is conferred to the behavior), euphoria, conflicts (with the self or others), time spent on the behavior, and relapse. Hausenblas and Downs (2002a) conceptualize EA as a multidimensional maladaptive pattern that generates a clinically significant emotional disturbance. Meanwhile, the DSM IV (American Psychiatric Association, 1994) identified EA based on the criteria of tolerance, withdrawal, intention effects, lack of control, time, reduction in other activities, and continuance. Currently, exercise addiction does not appear in international classifications of mental disorders, although the DSM 5 (American Psychiatric Association, 2013) considers some addiction disorders unrelated to substances, and it includes gambling addiction; in the chapter of disorders that require further study, it includes addiction to online gambling. For this reason, in the present study, we alternate the concepts of exercise addiction and excessive exercising as synonyms.

Muscle dysmorphia (MD) was initially described as “reverse anorexia” (Pope et al., 1997), given that the main symptoms are the belief of being too thin and not being muscular enough. The three criteria around which MD was supposed to be structured suggested that the individuals were (a) worried about the idea that their body did not have sufficient muscle, (b) generating significant clinical malaise, (c) and that their primary concern as well as their behavior could revolve around the idea of not being muscular enough. Unlike EA, MD is listed in the DSM 5 (American Psychiatric Association, 2013) as a specifier of body dysmorphic disorder (BDD), within the group of obsessive-compulsive disorder and related disorders. According to the American Psychiatric Association (2013), MD is defined as an individual's belief that his or her muscles are not big enough, and it can be delirious, depending on the level of seriousness and the lack of introspection. Some authors consider MD as a type of EA (Pace et al., 2020) within the framework of behavioral addictions, and they suggest that it should be included in the addiction model, in which an individual continues to follow a compulsive pattern of physical exercise and strict eating habits even though they have clear negative consequences, both psychological and physical, with the objective to maintain an ideal body (Foster et al., 2014; Griffiths et al., 2015). Specifically, the internalization of the ideal body image appears to be one of the strongest predictors of muscle dysmorphia (Schneider et al., 2016). Nevertheless, other authors believe that more research is needed before including MD within a model of body image addiction (Grant, 2015; Nieuwoudt, 2015). In this context, it is important to highlight the relationship between MD and BDD, where it has been found important correlations between appearance anxiety, BDD, and EA (Corazza et al., 2019).

Some studies show that there is an association between EA and MD. For example, Cafri et al. (2008) found that people with MD showed higher muscle dissatisfaction, appearance checking, and more bodybuilding dependence. Meanwhile, Babusa et al. (2015) confirmed that people with MD showed the highest levels of EA, exercising longer and more frequently in comparison with people without MD or with moderate risk factors. Olave et al. (2019) found that when MD increased, there was greater risk of EA, which favored muscle dissatisfaction and the exacerbation of MD.

Emotional dependence is also considered to be a behavioral addiction and is defined as the intense need for affection from one's partner (Castelló, 2005), even though it is not included within the various international classifications of mental and personality disorders. Some of the main characteristics are the idealization of one's partner, fear of loneliness and breakup, obsession with one's partner, low self-esteem, and submission (Castelló, 2005). Some studies refer to love addiction when talking about emotional dependence, and they compare it with classical substance addictions, because it involves craving to have a partner, deep worry when he or she is not with his or her partner, and intense suffering in the case of a breakup (Timmreck, 1990; Schaeffer, 1998).

Emotionally dependent individuals share certain characteristics with addictive pathologies, and they show difficulties with impulse control (Castelló, 2005; Villegas and Sánchez, 2013). Impulsivity, although it is not the main focus in this study, is a very important element that underlies addictive disorders and acts as a precursor of behavioral addictions (De Sola-Gutiérrez et al., 2013). Emotional dependence is also correlated with other behavioral addictions, such as sex addiction or compulsive sexual behavior (Sánchez Zaldívar and Iruarrizaga Díez, 2009; Iruarrizaga et al., 2019), Internet addiction, or cell phone addiction (Estévez et al., 2017).

The acquisition of dysfunctional childhood attachment is especially relevant in the etiology of emotional dependence. The development of childhood attachment (Ainsworth et al., 1978), where the socioemotional needs of children are not satisfied, is related to emotional dependence in adulthood, where affective deficiency lingers (Castelló, 2005), and romantic relationships are pursued as a means to meet emotional needs (Lemos and Londoño, 2006; Skvortsova and Shumskiy, 2014).

Estévez et al. (2018) point out that the relation between childhood attachment and impulsive behavior is mediated by attachment styles, and that certain attachment styles can be predictors of emotional dependence and can favor the continuity of abusive romantic relationships (Momeñe and Estévez, 2018). The style of parental socialization is also significantly related to teenagers' addictions. In a sample of young Spanish people, Martínez et al. (2013) found that lenient parenting acted as a protection factor against substance consumption in contrast to the authoritative style. Nevertheless, these results do not agree with research done in the United States, where authoritative parenting is presented as a protective factor and lenient parenting as a risk factor (Darling and Cumsille, 2003). Besides all this, attachment style is related to decision-making in such a way that insecure or anxious style is related to worse capacity in the executive process (Deniz, 2011), even emotional intelligence has been shown to mediate the relationship between secure attachment and decision-making (Phang et al., 2020). This provides information about the fact that people with maladaptive attachment styles may have reduced decision-making capacity, including those related to emotional regulation and opting for the compulsive practice of physical exercise as a strategy (Chamberlain and Grant, 2020).

Sex differences have been found, indicating that more men are affected by EA and MD (González-Cutre and Sicilia, 2012; Barrientos-Martínez et al., 2014). Although these problems are more prevalent among males, they are also found in women (Hale et al., 2013; Zeeck et al., 2018). In regard to emotional dependence, there are no clear differences between sex. Some studies do not find significant differences (Pradas and Perles, 2012; Momeñe et al., 2017; Urbiola et al., 2019), while others suggest that, in some emotional dependence subscales (Urbiola et al., 2017; Estévez et al., 2018), or even in total emotional dependence (Rodríguez-Pérez et al., 2019), males score higher.

In light of the lack of research relating EA and MD with other behavioral addictions, such as emotional dependence and its association with childhood attachment, the following objectives are set: (1) to analyze the sex differences found in emotional dependence, attachment dimensions, EA, and MD, (2) to analyze the association between EA and MD and other behavioral addictions, such as emotional dependence and attachment dimensions, and (3) to analyze the possible role that childhood attachment plays in mediating the interaction between emotional dependence and EA and MD.

## Materials and Methods

This is a cross-sectional, correlational, non-experimental study. The incidental sample was obtained from students of the Complutense University of Madrid and snowball sampling was used to recruit the participants. Information was gathered through pencil and paper surveys in the university classrooms. All the participants gave informed consent at the beginning of the study. The study has been carried out following the criteria of the Declaration of Helsinki (World Medical Association, 2013).

### Participants

The sample consisted of 366 participants (54.6% of women) between 17 and 31 years old (*M* = 23.53; S*D* = 6.48). Following are the most relevant sociodemographic data (see Table 1).

**Table 1:** Sociodemographic data of the participants.

**Variable**	**%**
Physical exercise in gym	54%
Spanish nationality	96%
University student	100%
Single	91.3%
Heterosexual	72.4%

### Measures

***Exercise addiction inventory*** (Terry et al., 2004). The Spanish adaptation conducted by Sicilia et al. (2013). It is a six-item self-reported survey with a five-point Likert-type scale response format (1 = *I completely disagree*; 5 = *I totally agree*). It establishes cutoff points to create classifications with respect to the risk of presenting exercise addiction or excessive exercise. In the present study, the level of internal consistency, measured through Cronbach's alpha, was 0.70.

***Muscle appearance satisfaction scale*** (Mayville et al., 2002). It is a self-reported instrument that informs about the cognitive, emotional, and behavioral aspects of muscle dysmorphia. It is formed by 19 items and 5 scales: (a) bodybuilding dependence, (b) muscle checking, (c) substance use, (d) injury, and (e) muscle satisfaction. Responses are collected on a five-point Likert scale (1 = *I completely disagree*; 5 = *I totally agree*). In this study, the adaptation conducted by González-Martí et al. (2012) was used, showing good psychometric properties with values for Cronbach's alpha between 0.86 and 0.95.

***Emotional dependence survey*** (Lemos and Londoño, 2006). This tool has been designed to evaluate emotional dependence in the Spanish-speaking population. It consists of 23 items and 6 factors: (a) separation anxiety, (b) partner's affective expression, (c) changing of plans, (d) fear of loneliness, (e) borderline expression, and (f) attention-seeking. Responses are reported on a six-point Likert scale (1 = *completely false about me; 6* = *this describes me perfectly*). It contains adequate psychometric properties, showing a Cronbach's alpha of 0.92 in the present study.

***CaMir-R*** (Pierrehumbert et al., 1996). This is a self-reported measure that evaluates representations of attachment and conceptions of family functioning. It has 32 items and 7 dimensions: (a) security, (b) preoccupation with family, (c) parental interference, (d) regard for authority, (e) parental permissiveness, (f) self-sufficiency and resentment toward parents, and (g) childhood trauma. Responses are reported on a five-point Likert scale (*1* = *strongly agree; 5* = *strongly disagree*). We have used the Spanish adaptation developed by Balluerka et al. (2011), which shows, in the present study, a Cronbach's alpha, which ranges between 0.60 and 0.85.

### Procedure

Students filled out the questionnaires in the classroom during teaching hours. They were informed that participation was voluntary, and that if they did not want to participate, they could give back a blank questionnaire without any penalty. Likewise, they were told that their data were confidential and anonymous and that they would only be used for statistical purposes. The students did not receive any kind of compensation. They were given between 15 and 20 min to fill out the questionnaires.

### Data Analysis

The analysis of the data was carried out with the IBM SPSS statistics software V25.0. Descriptive and correlational analyses were carried out between emotional dependence, attachment dimensions, exercise addiction, and muscle dysmorphia, although Pearson's *r*. Secondly, a stepwise multiple regression was carried out in order to analyze the predictive role of emotional dependence and attachment dimensions in exercise addiction and muscle dysmorphia. Next, a mediation analysis was carried out through regression analysis to check the role of attachment dimensions in mediating the relation between exercise addiction and emotional dependence. In addition, a second mediation analysis was carried out to study the role of attachment dimensions in mediating muscle dysmorphia and emotional dependence. Finally, the difference of means relative to sex was analyzed in emotional dependence, attachment dimensions, exercise addiction, and muscle dysmorphia through the Student's *t*-test. This way, the size of the difference of means was obtained through Cohen's *d* (1992), who pointed out that values under 0.20 would be considered small, around 0.50 would be considered medium, and, over 0.80, large.

## Results

### Sex Differences

In the first place, we analyzed the differences between emotional dependence, attachment dimensions, EA, and MD based on sex (see Table 2).

**Table 2:** Differences according to sex in emotional dependence, attachment, exercise addiction, and muscular dysmorphia.

**Variables**	**Women (*****n*** **=** **200)**		**Men (*****n*** **=** **166)**		***t***	**Effect size (d)**
	***M***	***DT***	***M***	***DT***		
**Emotional dependence**						
Changing of plans	6.58	3.08	8.23	3.69	4.61^**^	0.25
Borderline expression	3.90	1.67	4.25	1.90	1.86^*^	0.10
**Exercise addiction**						
Exercise addiction	12.55	5.03	14.59	5.99	3.48^*^	0.19
**Muscle dysmorphia**						
Muscle dysmorphia	31.63	7.65	37.07	13.43	4.63^**^	0.28
Substance use	4.66	1.47	5.73	2.94	4.31^**^	0.27
Exercise dependence	7.22	2.54	9.09	4.24	5.00^**^	0.30
Muscle satisfaction	8.30	2.92	8.98	2.58	2.39^*^	0.12
Muscle checking	5.87	2.35	7.43	4.10	4.36^**^	0.26
Injury	4.19	1.96	5.80	3.11	5.78^**^	0.33

* *p < 0.05;*

** *p < 0.001*.

Significant differences were observed between men and women, with males reporting higher scores in the subscales of emotional dependence, changing of plans, and borderline expression as well as in EA and MD, together with their five subscales. The effect sizes were small for borderline expression, EA, and muscle satisfaction, and moderate for the rest.

### Correlation Analyses Between Emotional Dependence, Attachment Dimensions, Exercise Addiction, and MD

Secondly, we analyzed Pearson's correlation between emotional dependence, attachment dimensions, exercise addiction, and MD (see Table 3). It was observed that EA showed a statistically significant positive correlation with emotional dependence and its six subscales (separation anxiety, partner's affective expression, changing of plans, fear of loneliness, borderline expression, and attention-seeking) with values between 0.16 and 0.28; and with MD and its subscales (substance use, muscle checking, injuries, and exercise dependence), presenting values between 0.42 and 0.70. Finally, with the attachment dimensions of preoccupation with family, parental interference, regard for authority and permissiveness, the correlation showed low values between 0.11 and 0.14. This showed that greater exercise addiction was indicative of higher levels of emotional dependence, MD, concern, interference and parental permissiveness, regard for authority, and self-sufficiency and resentment toward parents.

**Table 3:** Correlations between emotional dependence, exercise addiction, muscle dysmorphia, and attachment.

	**1**	**2**	**3**	**4**	**5**	**6**	**7**	**8**	**9**	**10**	**11**	**12**	**13**	**14**	**15**	**16**	**17**	**18**	**19**	**20**	**21**
Emotional dependence	–																				
Separation anxiety	0.92^**^	–																			
Partner's affective expression	0.88^**^	0.73^**^	–																		
Changing of plans	0.77^**^	0.67^**^	0.58^**^	–																	
Fear of loneliness	0.68^**^	0.52^**^	0.58^**^	0.36^**^	–																
Borderline expression	0.66^**^	0.55^**^	0.52^**^	0.48^**^	0.44^**^	–															
Attention seeking	0.77^**^	0.65^**^	0.69^**^	0.57^**^	0.46^**^	0.42^**^	–														
Exercise addiction	0.26^**^	0.24^**^	0.22^**^	0.18^**^	0.16^**^	0.20^**^	0.28^**^	–													
Muscle dysmorphia	0.35^**^	0.32^**^	0.28^**^	0.24^**^	0.24^**^	0.34^**^	0.34^**^	0.63^**^	–												
Muscle satisfaction	−0.15^**^	−0.14^**^	−0.18^**^	−0.04	−0.14^**^	−0.13^*^	−0.08	−0.04	−0.29^**^	–											
Substance use	0.20^**^	0.18^**^	0.13^*^	0.15^**^	0.10	0.28^**^	0.23^**^	0.42^**^	0.81^**^	−0.03	–										
Muscle checking	0.35^**^	0.31^**^	0.29^**^	0.24^**^	0.25^**^	0.30^**^	0.32^**^	0.51^**^	0.87^**^	−0.06	0.66^**^	–									
Injury	0.29^**^	0.26^**^	0.22^**^	0.22^**^	0.19^**^	0.26^**^	0.33^**^	0.62^**^	0.83^**^	−0.00	0.65^**^	0.69^**^	–								
Exercise dependence	0.30^**^	0.26^**^	0.22^**^	0.22^**^	0.21^**^	0.30^**^	0.29^**^	0.70^**^	0.89^**^	−0.03	0.72^**^	0.75^**^	0.75^**^	–							
Security	−0.05	−0.07	−0.01	0.01	−0.08	−0.11^*^	0.01	−0.03	−0.13^*^	0.04	−0.09	−0.09	−0.09	−0.14^**^	–						
Preoccupation with family	0.02	0.04	0.07	−0.02	0.00	−0.11^*^	−0.01	0.13^*^	0.05	−0.07	−0.03	0.05	0.03	0.06	0.17^**^	–					
Parental Interference	0.28^**^	0.27^**^	0.30^**^	0.14^**^	0.25^**^	0.16^**^	0.15^**^	0.11^*^	0.04	−0.15^**^	−0.07	0.04	0.01	0.02	0.05	0.24^**^	–				
Regard of authority	0.23^**^	0.25^**^	0.26^**^	0.13^*^	0.11^*^	0.09	0.17^**^	0.13^*^	0.17^**^	−0.13^*^	0.10	0.18^**^	0.11^*^	0.12^*^	0.20^**^	0.28^**^	0.34^**^	–			
Parental permissiveness	0.09	0.04	0.11^*^	0.08	0.09	0.04	0.14^*^	0.14^**^	0.07	0.13^*^	0.09	0.07	0.14^**^	0.10	0.22^**^	−0.02	0.03	0.02	–		
Self-sufficiency and resentment toward parents	0.39^**^	0.36^**^	0.38^**^	0.29^**^	0.24^**^	0.27^**^	0.29^**^	0.09	0.18^**^	−0.21^**^	0.09	0.17^**^	0.11^*^	0.09	0.06	0.17^**^	0.45^**^	0.42^**^	−0.03	–	
Childhood trauma	0.21^**^	0.20^**^	0.22^**^	0.09	0.18^**^	0.03	0.20^**^	0.07	0.08	−0.02	0.03	0.13^*^	0.05	0.06	0.26^**^	0.25^**^	0.22^**^	0.32^**^	0.15^**^	0.31^**^	–

* *p < 0.05;*

** *p < 0.001*.

Muscle dysmorphia is significantly and positively correlated with emotional dependence and its six subscales, with values ranging from 0.24 to 0.36; and with the attachment dimensions “regard for authority,” and “self-sufficiency and resentment toward parents.” It is negatively correlated with secure attachment, with values between −0.13 and −0.18. Emotional dependence showed statistically significant positive associations with the attachment dimensions “parental interference,” “regard for authority,” “self-sufficiency and resentment toward parents,” and “childhood trauma,” with values ranging between 0.11 and 0.35.

### The Predictive Role of Emotional Dependence and the Attachment Dimensions Over EA and MD

In the third place, we analyzed the predictive role of emotional dependence and the attachment dimensions over EA and MD (see Table 4).

**Table 4:** The predictive role of emotional dependence and attachment dimensions in exercise addiction and muscle dysmorphia.

**Predictor variable**	**B**	**β**	***T***	**Sig**.	**Criterion variable**
**EMOTIONAL DEPENDENCE**					**EXERCISE ADDICTION**
Attention seeking	0.56	0.23	3.06	0.00^**^	Exercise addiction
**ATTACHMENT**					
Preoccupation with family	0.30	0.15	2.72	0.01^*^	Exercise addiction
Parental permissiveness	0.37	0.13	2.52	0.01^*^	Exercise addiction
					(*R* = 0.36; *R*^2^ = 0.13; *R*^2^Corrected = 0.10)
**EMOTIONAL DEPENDENCE**					**MUSCLE DYSMORPHIA**
Borderline expression	1.42	0.23	3.81	0.00^**^	Muscle dysmorphia
Attention seeking	1.06	0.22	3.12	0.00^**^	Muscle dysmorphia
**ATTACHMENT**					
Security	−0.55	−0.15	−2.91	0.00^**^	Muscle dysmorphia
Parental interference	−0.44	−0.12	−2.09	0.04^*^	Muscle dysmorphia
Regard for authority	0.66	0.13	2.29	0.02^*^	Muscle dysmorphia
					(*R* = 0.46; *R*^2^ = 0.21; *R*^2^Corrected = 0.18)
**EMOTIONAL DEPENDENCE**					Muscle satisfaction
Partner's affective expression	−0.11	−0.18	−2.02	0.04^*^	Muscle satisfaction
**ATTACHMENT**					
Parental permissiveness	0.17	0.12	2.34	0.02^*^	Muscle satisfaction
Self-sufficiency and resentment toward parents	−0.12	−0.14	−2.16	0.03^*^	Muscle satisfaction
					(*R* = 0.31; *R*^2^ = 0.10; *R*^2^Corrected = 0.06)
**EMOTIONAL DEPENDENCE**					Muscle dependence
Borderline expression	0.44	0.22	3.60	0.00^**^	Muscle dependence
Attention seeking	0.34	0.22	3.04	0.00^**^	Muscle dependence
**ATTACHMENT**					
Security	−0.21	−0.18	−3.34	0.00^**^	Muscle dependence
Preoccupation with family	0.16	0.12	2.33	0.02^*^	Muscle dependence
Parental permissiveness	0.18	0.10	2.02	0.04^*^	Muscle dependence
					(*R* = 0.42; *R*^2^ = 0.18; *R*^2^Corrected = 0.15)
**EMOTIONAL DEPENDENCE**					Muscle checking
Borderline expression	0.32	0.17	2.74	0.01^*^	Muscle checking
Attention seeking	0.23	0.15	2.14	0.03^*^	Muscle checking
**ATTACHMENT**					
Security	−0.14	−0.12	−2.32	0.02^*^	Muscle checking
Parental interference	−0.14	−0.12	−2.14	0.03^*^	Muscle checking
Regard for authority	0.20	0.13	2.18	0.03^*^	Muscle checking
					(*R* = 0.42; *R*^2^ = 0.18; *R*^2^Corrected = 0.15)
**EMOTIONAL DEPENDENCE**					Substance use
Borderline expression	0.35	0.28	4.37	0.00^**^	Substance use
Attention seeking	0.21	0.21	2.86	0.00^**^	Substance use
**ATTACHMENT**					
Security	−0.08	−0.11	−2.07	0.04^*^	Substance use
Parental interference	−0.13	−0.17	−2.93	0.00^**^	Substance use
					(*R* = 0.38; *R*^2^ = 0.15; *R*^2^Corrected = 0.12)
**EMOTIONAL DEPENDENCE**					Injury
Borderline expression	0.23	0.15	2.47	0.01^*^	Injury
Attention seeking	0.33	0.28	3.87	0.00^**^	Injury
**ATTACHMENT**					
Security	−0.11	−0.13	−2.37	0.02^*^	Injury
Parental permissiveness	0.18	0.13	2.62	0.01^*^	Injury
					(*R* = 0.40; *R*^2^ = 0.16; *R*^2^Corrected = 0.13)

* *p < 0.05;*

** *p < 0.001*.

Attention-seeking and the attachment dimensions “preoccupation with family” and “parental permissiveness” predicted EA, explaining 36% of its variance. Likewise, borderline expression and regard for authority predicted MD, explaining 46% of its variance. Furthermore, affective expression toward the partner and the attachment dimensions “parental permissiveness,” and “self-sufficiency and resentment toward parents” predicted the 31% of variance in muscle satisfaction. In addition, borderline expression, attention-seeking and the attachment dimensions “security,” “preoccupation with family,” and “parental permissiveness” predicted exercise dependence. Borderline expression, attention-seeking, and the attachment dimensions “security,” “parental interference,” and “value to parental authority” predicted muscle checking, explaining 42% of its variance. Moreover, borderline expression, attention-seeking, and the attachment dimensions “security” and “parental interference” predicted 38% of the variance in substance use. Finally, the obtained results reflected that borderline expression, attention-seeking, and the attachment dimensions “security” and “parental permissiveness,” together, predicted 40% of the variance in injuries.

### The Role of Attachment Dimensions in Mediating the Association Between EA and Emotional Dependence

In the fourth place, we analyzed the role of attachment dimensions in mediating the association between EA and emotional dependence (see Table 5).

**Table 5:** Mediation of attachment dimensions in the relationship between exercise addiction and emotional dependence.

**Independent variable**	**Dependent variable**	**Total effect**	**Direct effect**	**Bootstrap 95% CI**	
Exercise addiction	Emotional dependence	0.88^**^	0.70^**^	0.36	1.05
Exercise addiction	Separation anxiety	0.33^**^	0.26^**^	0.12	0.39
Exercise addiction	Partner's affective expression	0.18^**^	0.12^**^	0.04	0.21
Exercise addiction	Changing of plans	0.10^**^	0.09^**^	0.02	0.16
Exercise addiction	Fear of loneliness	0.09^**^	0.07^*^	0.01	0.12
Exercise addiction	Attention seeking	0.11^**^	0.10^**^	0.06	0.14

* *p < 0.05;*

** *p < 0.001*.

We found that 20.45% of the association between EA and emotional dependence was explained by the attachment dimensions. Likewise, 21.21% of the association between EA and separation anxiety was explained by the attachment dimensions. About 33.33% of the association between EA and borderline expression was explained by the attachment dimensions. Ten percent of the association between EA and changing of plans was explained by the attachment dimensions. About 22.22% of the association between EA and fear of loneliness was explained by the attachment dimensions. Finally, 9.09% of the association between EA and attention-seeking was explained by the attachment dimensions.

### The Role of the Attachment Dimensions in Mediating the Association Between MD and Emotional Dependence

Last, we analyzed the role of the attachment dimensions in mediating the association between MD and emotional dependence (see Table 6). This was carried out through a linear regression analysis.

**Table 6:** Mediation of attachment styles in the relationship between muscle dysmorphia and emotional dependence.

**Independent variable**	**Dependent variable**	**Total effect**	**Direct effect**	**Bootstrap 95% CI**	
Muscle dysmorphia	Emotional dependence	0.60^**^	0.47^**^	0.30	0.64
Muscle dysmorphia	Separation anxiety	0.22^**^	0.16^**^	0.09	0.24
Muscle dysmorphia	Partner's affective expression	0.12^**^	0.09^**^	0.05	0.13
Muscle dysmorphia	Changing of plans	0.08^**^	0.06^**^	0.03	0.09
Muscle dysmorphia	Fear of loneliness	0.07^**^	0.05^**^	0.03	0.08
Muscle dysmorphia	Attention seeking	0.07^**^	0.06^**^	0.03	0.08
Muscle satisfaction	Emotional dependence	−1.03^**^	−0.53	−1.17	0.14
Muscle satisfaction	Separation anxiety	−0.38^*^	−0.16	−0.44	0.10
Muscle satisfaction	Partner's affective expression	−0.30^**^	−0.19^*^	−0.36	−0.03
Muscle satisfaction	Fear of loneliness	−0.16^*^	−0.11	−0.22	−0.00
Muscle satisfaction	Borderline expression	−0.08^*^	−0.05	−0.11	0.01
Muscle checking	Emotional dependence	1.95^**^	1.52^**^	0.93	2.07
Muscle checking	Separation anxiety	0.70^**^	0.53^**^	0.29	0.74
Muscle checking	Partner's affective expression	0.39^**^	0.29^**^	0.18	0.41
Muscle checking	Changing of plans	0.25^**^	0.20^**^	0.09	0.30
Muscle checking	Fear of loneliness	0.23^**^	0.18^**^	0.09	0.28
Muscle checking	Borderline expression	0.16^**^	0.14^**^	0.09	0.19
Muscle checking	Attention seeking	0.22^**^	0.17^**^	0.10	0.24
Substance use	Emotional dependence	1.62^**^	1.30^**^	0.45	2.21
Substance use	Separation anxiety	0.57^**^	0.45^**^	0.14	0.77
Substance use	Partner's affective expression	0.25^*^	0.17	0.02	0.35
Substance use	Changing of plans	0.23^**^	0.18^*^	0.04	0.32
Substance use	Borderline expression	0.21^**^	0.20^**^	0.12	0.29
Substance use	Attention seeking	0.23^**^	0.19^**^	0.08	0.30
Injury	Emotional dependence	2.04^**^	1.64^**^	0.90	2.34
Injury	Separation anxiety	0.72^**^	0.57^**^	0.26	0.86
Injury	Partner's affective expression	0.37^**^	0.27^**^	0.11	0.43
Injury	Changing of plans	0.29^**^	0.24^**^	0.11	0.38
Injury	Fear of loneliness	0.21^**^	0.17^**^	0.05	0.29

* *p < 0.05;*

** *p < 0.001*.

About 21.67% of the association between MD and emotional dependence, 27.27% of the association between MD and separation anxiety, 25% of the association between MD and affective expression toward the partner, 25% of the association between MD and changing of plans, 28% of the association between MD and fear of loneliness, and 14.28% of the association between MD and attention-seeking was explained by the attachment dimensions. Furthermore, 36.67% of the association between muscle satisfaction and affective expression toward the partner was explained by the attachment dimensions. Likewise, the results showed that 22.05% of the association between muscle checking and emotional dependence, 24.98% of the association between muscle checking and separation anxiety, 25.64% of the association between muscle checking and affective expression toward the partner, 20% of the association between muscle checking and changing of plans, 21.73% of the association between muscle checking and fear of loneliness, 12.50% of the association between muscle checking and borderline expression, and 22.72% of the association between muscle checking and attention-seeking were explained by the attachment dimensions. In addition, 19.75% of the association between substance use and emotional dependence, 21.05% of the association between substance use and separation anxiety, 21.73% of the association between substance use and changing of plans, 4.73% of the association between substance use and borderline expression, and 17.39% of the association between substance use and attention-seeking were explained by the attachment dimensions. Finally, 19.61% of the association between injuries and emotional dependence, 20.83% of the association between injuries and separation anxiety, 27.02% of the association between injuries and affective expression toward the partner, 17.24% of the association between injuries and changing of plans, 19.04% of the association between injuries and fear of loneliness, 11.76% of the association between injuries and borderline expression, and 14.28% of the association between injuries and attention-seeking were explained by the attachment dimensions.

## Discussion

Significant differences occur based on sex. Males score higher in the subscales of emotional dependence, changing of plans, and borderline expression. These results match with previous results, where men showed higher emotional dependence than women (Urbiola and Estévez, 2015; Urbiola et al., 2017). This means that men are more likely to quit other activities and stop seeing people to be with their partners and spend as much time as possible with them and that they will exhibit borderline behaviors when expressing their emotions toward their partners. Significant differences are also found in favor of men in EA and MD, together with their five subscales. These results also coincide with the data shown in other studies (Hausenblas and Downs, 2002b; González-Cutre and Sicilia, 2012; Compte and Sepúlveda, 2014).

This study manifests the statistically significant association between EA or excessive exercising and MD. The results coincide with those encountered in other studies where both constructs appear related. Hale et al. (2013) found that people that practice bodybuilding show MD and EA more often than those who are lifting weights recreationally. In a sample of athletes, Latorre Román et al. (2016) found that EA was significantly associated with body dissatisfaction, a relevant aspect of MD. Even though causal relationships cannot be established, it seems coherent to think that a person with MD develops comorbidity with EA, given that they build their muscles through physical exercise. What is not as clear is that a person with EA will necessarily develop MD. However, as pointed out in Olave et al. (2019), a higher risk of EA is associated with an increase in muscle dissatisfaction, and, as a consequence, an increase in MD. Corazza et al. (2019) also found an important relation between MD and EA, and also BDD and appearance anxiety in people who exercise in the gym.

EA also appears to be associated with emotional dependence. These results are relevant because they increase the evidence that behavioral addictions are interrelated. In previous studies, EA was related to shopping addiction and food addiction (Lejoyeux et al., 2008), but not found to be related to emotional dependence. In this sense, the role of impulsiveness is probably fundamental, given that it underlies addictive pathologies (De Sola-Gutiérrez et al., 2013). An explanatory hypothesis could be that the person with emotional dependence, in his or her pursuit not to lose the beloved person, might exercise his or her body to achieve a desired body figure and feel attractive, which could lead to compulsive physical exercise. In respect of its relationship with the attachment dimensions, EA has been related to family concern, parental interference, regard for authority, and permissiveness. These attachment dimensions are insecure, and, except for permissiveness, they are marked by an anxiogenic component. These results contradict the ones found by Lichtenstein et al. (2014), who did not find significant results in the attachment style in a sample of people with EA, although they did find a certain tendency to the insecure attachment dimension.

Muscle dysmorphia is significantly associated with EA and emotional dependence. It is not surprising that individuals with emotional dependence also show MD, given that both problems are closely related to low self-esteem and a negative evaluation of oneself, which would lead to body dissatisfaction and compulsive physical exercise. Furthermore, MD has been found to be associated with maladaptive attachment dimensions, which means that as MD increases so do emotional dependence and the attachment dimensions “regard for authority” and “self-sufficiency” and “resentment toward parents,” while secure attachment decreases. These findings are consistent with Fabris et al. (2018), who found a significant association between having an insecure attachment type (avoidant) and the risk of developing MD. It is also necessary to point out the importance of substance use, part of DM, and its relationship with full MD and EA. Mooney et al. (2017) found in a sample of gym and fitness club users a risk of EA, appearance anxiety, and low self-esteem, as well as an inclination to use products to supplement their physical exercise protein (41%), caffeine (30%), nitric oxide (13%), and anabolic steroids (7%). In this same line, Dores et al. (2021) found in a large sample, including the participants from Spain (15.7%), during the lockdown because the COVID-19, strong relations between physical exercise and use of image and performance-enhancing drugs, and, as in other studies, anxiety about appearance also increased the probability of using these drugs.

The connection between emotional dependence and attachment dimension has also become evident. Thus, with greater emotional dependence, there would be greater parental interference, regard for authority, self-sufficiency, and resentment toward parents, and childhood trauma. These results coincide with the results of other studies (Estévez et al., 2018), where it was found that parental permissiveness, self-sufficiency, resentment toward parents, and childhood trauma predicted emotional dependence. This suggests that individuals with emotional dependence are more likely to show an insecure attachment style in their interpersonal relationships.

We must highlight the mediatory role of attachment, given that it explains part of the connection between EA and MD and emotional dependence, especially borderline expression. This component of emotional dependence refers to the expression of emotions in an uncontrolled way, as though they had borderline personality disorder (BPD). Contrasting evidence exists that individuals with BPD are more likely to have an insecure attachment style (Gunderson, 1996; Nickell et al., 2002; Bakermans-Kranenburga and van Ijzendoorna, 2009; Mosquera and González, 2013). This result coincides with those of Estévez et al. (2018), who examined the mediatory role of attachment in connection with emotional dependence and impulsive behaviors.

The predictive role of emotional dependence in EA and MD has been confirmed. People who show the need to attract their partners' attention and borderline expression of their emotions will be more likely to develop EA and/or MD. The same happens with insecure attachment styles. However, secure attachment style also predicts some of the scales of MD, which suggests that even people with secure attachment can experience exercise dependence, constantly check their muscles in front of the mirror, use substances to increase their muscles, and persist with their physical exercise despite the occurrence of injuries.

### Practical Implications

These results bring evidence of the existing connections between EA, emotional dependence, MD, and some insecure attachment styles in people that exercise in and out of the gym. The dissemination of these findings is necessary so that professionals in the field of mental health and sport science can conduct prevention and be better prepared to intervene with individuals with EA and MD. It has been proved that excessive physical exercise can increase the likelihood of addictive behaviors and health problems (Nogueira et al., 2018). The current treatments for EA are oriented toward a cognitive-behavioral intervention (Márquez and Vega, 2015), and for MD pharmacologist treatment, cognitive behavioral therapy, and metacognitive therapy (Nicewicz and Boutrouille, 2020). In addition to this, it will be necessary to explore and work on attachment style, interpersonal connections, and comorbidity with other addictive pathologies, such as emotional dependence. On the other hand, these dependencies could be partly reduced by improving emotional regulation skills, since this would reduce impulsive conduct, worries; improve coping skills, decision-making, and the aversiveness of the situations (Gómez-Romero et al. 2020); and could cultivate secure attachment.

Furthermore, incorporating elements of emotional regulation in the practice of sport and physical exercise, as well as in the education field, favored the decrease of these addictions. In this sense, adolescence would be a critical stage, where physical changes occur as well as the first social and sexual relationships. Programs designed to prevent these addictions could be very beneficial for adult relationships and for the acceptance of one's body.

### Limitations

The present study has some limitations. It is cross-sectional, which means that causality connections cannot be established and conclusions are limited. In future investigations, longitudinal studies should be carried out in order to validate the predictive role of the analyzed variables and include other psychological variables, such as emotional regulation or impulsivity, which can intervene in the connection between these variables. Other limitations are found in the evaluation tools, given that they are self-reported measures, and they are screening tools with no diagnostic validity (Sicilia et al., 2015). Also, in this study, statistical analyses have been carried out in the general population, which is why the next research objective is to study these variables in a population with EA and DM and observe if there are differences with control participants. Another challenge for the future is to include the variable of BDD, since it is closely related to DM and EA (Corazza et al., 2019), and observe if it interacts with the variables studied. Finally, because the sample consists of university students, the generalization of the results to the general population is limited.

## Data Availability Statement

The raw data supporting the conclusions of this article will be made available by the authors, without undue reservation.

## Ethics Statement

This work is part of the research for the doctoral thesis, and has been approved by the doctoral committee of the Faculty of Psychology of the Complutense University of Madrid. The patients/participants provided their written informed consent to participate in this study. This study has been approved by the deontological commission of the Faculty of Psychology of the Complutense University of Madrid.

## Author Contributions

LO has carried out the data collection and has written the manuscript. II has directed the project and has written the manuscript. AE, JM, and RM-N have performed the data analysis. MG-R and MB have done the translation into English. All authors assisted with the preparation of the manuscript.

## Conflict of Interest

The authors declare that the research was conducted in the absence of any commercial or financial relationships that could be construed as a potential conflict of interest.

## References

[B1] Ainsworth M. D. S. Blehar M. C. Waters E. Wall S. (1978). Patterns of Attachment: A Psychological Study of the Strange Situation. Routledge: Taylor y Francis Group.

[B2] American Psychiatric Association (1994). Diagnostic and Statistical Manual of Mental Disorders, 4th Edn. Masson.

[B3] American Psychiatric Association (2013). Diagnostic and Statistical Manual of Mental Disorders (DSM-5). American Psychiatric Publishing.

[B4] Babusa B. Czeglédi E. Túry F. Mayville S. B. Urbán R. (2015). Differentiating the levels of risk for muscle dysmorphia among Hungarian male weightlifters: a factor mixture modeling approach. Body Image. 12, 14–21. 10.1016/j.bodyim.2014.09.001 25280243

[B5] Bakermans-Kranenburga M. van Ijzendoorna M. (2009). The first 10,000 adult attachment interviews: distributions of adult attachment representations in clinical and non-clinical groups. Attachment Human Dev. 11, 223–263. 10.1080/14616730902814762 19455453

[B6] Balluerka N. Lacasa F. Gorostiaga A. Muela A. Pierrehumbert B. (2011). Versión reducida del cuestionario CaMir (CaMir-R) para la evaluación del apego. Psicothema 23, 486–494. 21774904

[B7] Barrientos-Martínez N. Escoto C. Bosques L. E. Enríquez-Ibarra J. Juárez-Lugo C. S. (2014). Interiorización de ideales estéticos y preocupación corporal en hombres y mujeres usuarios de gimnasio. Rev. Mexicana Trastornos Alimentarios 5, 29–38. 10.1016/s2007-1523(14)70374-x

[B8] Cafri G. Olivardia R. Thompson J. K. (2008). Symptom characteristics and psychiatric comorbidity among males with muscle dysmorphia. Comprehens. Psychiatry 49, 374–379. 10.1016/j.comppsych.2008.01.003 18555058

[B9] Castelló J. (2005). Dependencia Emocional: Caracterí*sticas y Tratamiento*. Alianza.

[B10] Chamberlain S. R. Grant J. E. (2020). Is problematic exercise really problematic? A dimensional approach. CNS Spectrums 25, 64–70. 10.1017/S1092852919000762 30915933 PMC7002162

[B11] Compte E. J. Sepúlveda A. R. (2014). Dismorfia muscular: perspectiva histórica y actualización en su diagnóstico, evaluación y tratamiento. Behav. Psychol. Psicol. Conductual 22, 307–326. Available online at: https://www.behavioralpsycho.com/producto/dismorfia-muscular-perspectiva-historica-y-actualizacion-en-su-diagnostico-evaluacion-y-tratamiento/

[B12] Corazza O. Simonato P. Demetrovics Z. Mooney R. van de Ven K. Roman-Urresterazu A.. (2019). The emergence of exercise addiction, body dysmorphic disorder, and other image-related psychopathological correlates in fitness settings: a cross sectional study. PLoS ONE 14:e0213060. 10.1371/journal.pone.0213060 30943200 PMC6447162

[B13] Darling N. Cumsille P. (2003). Theory, measurement, and methods in the study of family influences on adolescent smoking. Addiction 98, 21–36. 10.1046/j.1360-0443.98.s1.3.x 12752360

[B14] De Sola-Gutiérrez J. Rubio-Valladolid G. Rodríguez de Fonseca F. (2013). La impulsividad: Antesala de las adicciones comportamentales? Salud Drogas 13, 145–155. 10.21134/haaj.v13i2.211

[B15] Deniz M. (2011). An investigation of decision making styles and the five-factor personality traits with respect to attachment styles. Educ. Sci. Theory Prac. 11, 105–113.

[B16] Dores A. R. Carvalho I. P. Burkauskas J. Simonato P. De Luca I. Mooney R.. (2021). Exercise and use of enhancement drugs at the time of the COVID-19 pandemic: a multicultural study on coping strategies during self-isolation and related risks. Front. Psychiatry 12:165. 10.3389/fpsyt.2021.648501 33776822 PMC7988429

[B17] Echeburúa E. Labrador F. J. Becoña E. (2009). Adicción a las Nuevas Tecnologías en Jóvenes y Adolescentes. Pirámide.

[B18] Estévez A. Chávez-Vera M. D. Momeñe J. Olave L. Vázquez D. Iruarrizaga I. (2018). El papel de la dependencia emocional en la relación entre el apego y la conducta impulsiva. Annal. Psychol. 34, 438–445. 10.6018/analesps.34.3.313681

[B19] Estévez A. Urbiola I. Iruarrizaga I. Onaindia J. Jauregui P. (2017). Dependencia emocional y consecuencias psicológicas del abuso de internet y móvil en jóvenes. Ann. Psychol. 33, 260–268. 10.6018/analesps.33.2.255111

[B20] Fabris M. A. Longobardi C. Prino L. E. Settanni M. (2018). Attachment style and risk of muscle dysmorphia in a sample of male bodybuilders. Psychol. Men Masculinity 19, 273–281. 10.1037/men0000096

[B21] Foster A. Shorter G. Griffiths M. (2014). Muscle dysmorphia: could it be classified as an addiction to body image? J. Behav. Addict. 4, 1–5. 10.1556/JBA.3.2014.001 25592218 PMC4394845

[B22] González-Cutre D. Sicilia Á. (2012). Dependencia del ejercicio físico en usuarios españoles de centros de acondicionamiento físico (fitness): diferencias según el sexo, la edad y las actividades practicadas. Behav. Psychol. Psicol. Conductual 20, 349–364.

[B23] González-Martí I. Bustos J. G. F. Jordán O. R. C. Mayville S. B. (2012). Validation of a Spanish version of the Muscle Appearance Satisfaction Scale: Escala de Satisfacción Muscular. Body Image 9, 517–523. 10.1016/j.bodyim.2012.05.002 22682995

[B24] Grant J. (2015). Commentary on: muscle dysmorphia: could it be classified as an addiction to body image? J. Behav. Addict. 4, 6–7. 10.1556/JBA.3.2014.021 25592216 PMC4394846

[B25] Griffiths M. Foster A. Shorter G. (2015). Commentary on muscle dysmorphia as an addiction: a response to Grant (2015) and Nieuwoudt (2015). J. Behav. Addict. 4, 11–13. 10.1556/JBA.4.2015.1.4 25786494 PMC4394848

[B26] Griffiths M. D. (1997). Exercise addiction: a case study. Addict. Res. 5, 161–168. 10.3109/16066359709005257

[B27] Gunderson J. G. (1996). Borderline patient's intolerance of aloneness: insecure attachments and therapist availability. Am. J. Psychiatry 153, 752–758. 10.1176/ajp.153.6.752 8633685

[B28] Hale B. D. Diehl D. Weaver K. Briggs M. (2013). Exercise dependence and muscle dysmorphia in novice and experienced female bodybuilders. J. Behav. Addict. 2, 244–248. 10.1556/JBA.2.2013.4.8 25215207 PMC4154574

[B29] Hausenblas H. A. Downs D. S. (2002a). How much is too much? The development and validation of the exercise dependence scale. Psychol. Health 17, 387–404. 10.1080/0887044022000004894

[B30] Hausenblas H. A. Downs D. S. (2002b). Relationship among sex, imagery, and exercise dependence symptoms. Psychol. Addict. Behav. 16, 169–172. 10.1037/0893-164X.16.2.169 12079258

[B31] Iruarrizaga I. Estévez A. Momeñe J. Olave L. Fernández-Cárdaba L. Chávez-Vera M. D.. (2019). Dificultades en la regulación emocional, esquemas inadaptados tempranos, y dependencia emocional en la adicción al sexo o comportamiento sexual compulsivo en la adolescencia. Rev. Española Drogodependencias 44, 76–103.

[B32] Latorre Román P. Á. Jiménez Obra A. Párraga Montilla J. García Pinillos F. (2016). Dependencia al ejercicio físico e insatisfacción corporal en diferentes deportes de resistencia y su relación con la motivación al deporte. Revista de psicología del deporte. 25, 113–120. Available online at: https://www.redalyc.org/articulo.oa?id=235143645015

[B33] Lejoyeux M. Avril M. Richoux C. Embouazza H. Nivoli F. (2008). Prevalence of exercise dependence and other behavioral addictions among clients of a Parisian fitness room. Comprehens. Psychiatry 49, 353–358. 10.1016/j.comppsych.2007.12.005 18555055

[B34] Lemos M. Londoño N. H. (2006). Construcción y validación del cuestionario de dependencia emocional en población colombiana. Acta Colombiana Psicol. 9, 127–140.

[B35] Lichtenstein M. B. Christiansen E. Elklit A. Bilenberg N. Støving R. K. (2014). Exercise addiction: a study of eating disorder symptoms, quality of life, personality traits and attachment styles. Psychiatry Res. 215, 410–416. 10.1016/j.psychres.2013.11.010 24342179

[B36] Márquez S. Vega R. D. L. (2015). La adicción al ejercicio: un trastorno emergente de la conducta. Nutr. Hospital. 31, 2384–2391. 10.3305/nh.2015.31.6.8934 26040342

[B37] Martínez I. Fuentes M. C. García F. Madrid I. (2013). El estilo de socialización familiar como factor de prevención o riesgo para el consumo de sustancias y otros problemas de conducta en los adolescentes españoles. Adicciones 25:235. 10.20882/adicciones.51 23880836

[B38] Mayville S. B. Williamson D. A. White M. A. Netemeyer R. G. Drab D. L. (2002). Development of the Muscle Appearance Satisfaction Scale: a self-report measure for the assessment of muscle dysmorphia symptoms. Assessment 9, 351–360. 10.1177/1073191102238156 12462755

[B39] Momeñe J. Estévez A. (2018). Los estilos de crianza parentales como predictores del apego adulto, de la dependencia emocional y del abuso psicológico en las relaciones de pareja adultas. Behav. Psychol. Psicol. Conductual 26, 359–378.

[B40] Momeñe J. Jáuregui P. Estévez A. (2017). El papel predictor del abuso psicológico y la regulación emocional en la dependencia emocional. Psicol. Conductual 25:65.

[B41] Mooney R. Simonato P. Ruparelia R. Roman-Urrestarazu A. Martinotti G. Corazza O. (2017). The use of supplements and performance and image enhancing drugs in fitness settings: a exploratory cross-sectional investigation in the United Kingdom. Human Psychopharmacol. Clin. Exp. 32:e2619. 10.1002/hup.2619 28657184

[B42] Mosquera D. González A. (2013). Del apego temprano a los síntomas del trastorno límite de personalidad. Rev. Digital Med. Psicosomática Psicoter. 3, 1–33.

[B43] Nicewicz H. R. Boutrouille J. F. (2020). Body Dysmorphic Disorder (BDD, Dysmorphobia, Dysmorphic Syndrome). StatPearls Publishing. Available online at: https://www.ncbi.nlm.nih.gov/books/NBK555901/32310361

[B44] Nickell A. D. Waudby C. J. Trull T. J. (2002). Attachment, parental bonding, and Borderline Personality Disorder features in young adults. J. Personality Disord. 16, 148–159. 10.1521/pedi.16.2.148.22544 12004491

[B45] Nieuwoudt J. E. (2015). Commentary on: muscle dysmorphia: could it be classified as an addiction to body image?. J. Behav. Addict. 4, 8–10. 10.1556/JBA.3.2014.020 25592217 PMC4394847

[B46] Nogueira A. Molinero O. Salguero A. Márquez S. (2018). Exercise addiction in practitioners of endurance sports: a literature review. Front. Psychol. 9:1484. 10.3389/fpsyg.2018.01484 30174636 PMC6107830

[B47] Olave L. Momeñe J. Rodríguez Pérez J. G. Estévez A. I. Iruarrizaga I. (2019). ¿Existen diferencias en los esquemas cognitivos y en la autoestima entre las personas con adicción al ejercicio físico o con dismorfia muscular? Rev. Española Drogodependencias. 44, 43–61. Available online at: https://www.aesed.com/upload/files/v44n2_art2-dismorfia.pdf

[B48] Pace U. D'Urso G. Passanisi A. Mangialavori S. Cacioppo M. Zappulla C. (2020). Muscle dysmorphia in adolescence: the role of parental psychological control on a potential behavioral addiction. J. Child Family Stud. 29, 455–461. 10.1007/s10826-019-01547-w

[B49] Phang A. Fan W. Arbona C. (2020). Secure attachment and career indecision: the mediating role of emotional intelligence. J. Career Dev. 47, 657–670. 10.1177/0894845318814366

[B50] Pierrehumbert B. Karmaniola A. Sieye A. Meister C. Miljkovitch R. Halfon O. (1996). Les modèles de relations: développement d'un auto-questionnaire d'attachement pour adultes. Psychiatrie l'Enfant 39, 161–206.

[B51] Pope H. G. Gruber A. J. Choi P. Olivardia R. Phillips K. A. (1997). Muscle dysmorphia: an underrecognized form of body dysmorphic disorder. Psychosomatics 38, 548–557. 10.1016/S0033-3182(97)71400-2 9427852

[B52] Pradas E. Perles F. (2012). Resolución de conflictos de pareja en adolescentes, sexismo y Emotionaldependence. Quaderns Psicol. 14, 45–60. 10.5565/rev/qpsicologia.1041

[B53] Rodríguez-Pérez J. G. Momeñe J. Olave L. Estévez A. Iruarrizaga I. (2019). La dependencia emocional y la resolución de conflictos en heterosexuales, homosexuales y bisexuales. Rev. Española Drogodependencias. 44, 59–79. Available online at: https://www.aesed.com/upload/files/v44n1_art4.pdf

[B54] Sánchez Zaldívar S. Iruarrizaga Díez I. (2009). Nuevas dimensiones, nuevas adicciones: la adicción al sexo en internet. Psychosocial Intervent. 18, 255–268. 10.5093/in2009v18n3a6

[B55] Schaeffer B. (1998). ¿Es Amor o es adicción?Apóstrofe. Barcelona: Apóstrofe.

[B56] Scharmer C. Gorrell S. Schaumberg K. Anderson D. (2020). Compulsive exercise or exercise dependence? Clarifying conceptualizations of exercise in the context of eating disorder pathology. Psychol. Sport Exerc. 46:101586. 10.1016/j.psychsport.2019.101586 34093941 PMC8174529

[B57] Schneider C. Rollitz L. Voracek M. Hennig-Fast K. (2016). Biological, psychological, and sociocultural factors contributing to the drive for muscularity in weight-training men. Front. Psychol. 7:1992. 10.3389/fpsyg.2016.01992 28066308 PMC5174100

[B58] Sicilia A. Alías-García A. Ferriz R. Moreno-Murcia J. A. (2013). Spanish adaptation and validation of the Exercise Addiction Inventory (EAI). Psicothema 25, 377–383. 10.7334/psicothema2013.21 23910754

[B59] Sicilia A. González-Cutre D. Férriz R. Lirola M. J. (2015). “Adicción al ejercicio físico,” in Motricidad Humana. Hacia una vida más Saludable, eds Moreno Murcia J. A. de Souza Costa Concesiçao M. C. (Universidad Central de Chile), 249–274.

[B60] Skvortsova S. N. Shumskiy V. B. (2014). Existential-phenomenological analysis of dependence in close interpersonal relationships. Existenzanalyse 31, 4–13.

[B61] Terry A. Szabo A. Griffiths M. (2004). The exercise addiction inventory: a new brief screening tool. Addict. Res. Theory 12, 489–499. 10.1080/16066350310001637363

[B62] Timmreck T. C. (1990). Overcoming the loss of a love: preventing love addiction and promoting positive emotional health. Psycho. Rep. 66, 515–528. 10.2466/pr0.1990.66.2.515 2190254

[B63] Urbiola I. Estévez A. Iruarrizaga I. Jauregui P. (2017). Dependencia emocional en jóvenes: relación con la sintomatología ansiosa y depresiva, autoestima y diferencias de género. Ansiedad y estrés 23, 6–11. 10.1016/j.anyes.2016.11.003

[B64] Urbiola I. Estévez A. Iruarrizaga I. Momeñe J. Bilbao P. J. Bilbao M.. (2019). Dependencia emocional en el noviazgo: papel mediador entre la autoestima y la violencia psicológica en jóvenes. Rev. Española Drogodependencias. 44, 13–27. Available online at: https://www.aesed.com/upload/files/v44n1_art1.pdf

[B65] Urbiola I. Estévez A. (2015). Dependencia emocional y esquemas desadaptativos tempranos en el noviazgo de adolescentes y jóvenes. Behav. Psychol. Psicol. Conductual 23, 571–587.

[B66] Villegas M. J. Sánchez N. (2013). Dependencia emocional en un grupo de mujeres denunciantes de maltrato de pareja. Textos Sentidos 7, 10–29.

[B67] World Medical Association (2013). World Medical Association, Declaration of Helsinki: ethical principles for medical research involving human subjects. JAMA 310:21. 10.1001/jama.2013.281053 24141714

[B68] Zeeck A. Welter V. Alatas H. Hildebrandt T. Lahmann C. Hartmann A. (2018). Muscle Dysmorphic Disorder Inventory (MDDI): validation of a German version with a focus on gender. PLoS ONE 13:e0207535. 10.1371/journal.pone.0207535 30444914 PMC6239320

